# Genome-Wide Identification of the Odorant Receptor Gene Family and Revealing Key Genes Involved in Sexual Communication in *Anoplophora glabripennis*

**DOI:** 10.3390/ijms24021625

**Published:** 2023-01-13

**Authors:** Sainan Zhang, Meng Li, Yabei Xu, Yuxuan Zhao, Yiming Niu, Shixiang Zong, Jing Tao

**Affiliations:** Beijing Key Laboratory for Forest Pest Control, Beijing Forestry University, Beijing 100083, China

**Keywords:** *Anoplophora glabripennis*, genome-wide analysis, odorant receptors, sexual communication, expression profile, olfactory recognition

## Abstract

Insects use a powerful and complex olfactory recognition system to sense odor molecules in the external environment to guide behavior. A large family of odorant receptors (ORs) mediates the detection of pheromone compounds. *Anoplophora glabripennis* is a destructive pest that harms broad-leaved tree species. Although olfactory sensation is an important factor affecting the information exchange of *A. glabripennis*, little is known about the key *ORs* involved. Here, we identified ninety-eight *AglaORs* in the Agla2.0 genome and found that the *AglaOR* gene family had expanded with structural and functional diversity. RT-qPCR was used to analyze the expression of *AglaORs* in sex tissues and in adults at different developmental stages. Twenty-three *AglaORs* with antennal-biased expression were identified. Among these, eleven were male-biased and two were female-biased and were more significantly expressed in the sexual maturation stage than in the post-mating stage, suggesting that these genes play a role in sexual communication. Relatively, two female-biased *AglaORs* were overexpressed in females seeking spawning grounds after mating, indicating that these genes might be involved in the recognition of host plant volatiles that may regulate the selection of spawning grounds. Our study provides a theoretical basis for further studies into the molecular mechanism of *A. glabripennis* olfaction.

## 1. Introduction

Insects have evolved a special olfactory system that can detect volatile substances in the air with specificity, providing accurate information about the environment to regulate behaviors such as feeding, mating, and egg-laying [1,2]. After the liposolubility odor molecules in the external environment enter the sensillum lymph of water solubility through the micropores of the insect sensillum epidermis, firstly, odorant-binding proteins (OBPs) and chemosensory proteins (CSPs) recognize and bind the odor molecules, and also assist odor molecules in transporting them to the periphery of the dendritic membrane of olfactory neurons (ORNs) and ultimately activate receptors. Secondly, after the receptors convert chemical signals into electrophysiological signals and transmit them to the central nervous system of insects for integration, the brain issues instructions to guide insects to conduct physiological reactions. [3,4]. Insect receptors include three gene families: odorant receptors (ORs) that are sensitive to alcohols, ketones, and esters; ionotropic receptors (IRs) that sense amines and acids; and gustatory receptors (GRs) that sense soluble chemicals [5,6,7]. As ligand-gated ion channels involved in odor recognition and signal transduction, ORs can specifically recognize different host volatiles and pheromone molecules [8]. In 1991, the first OR was identified in the mammal Rattus norvegicus, after which the first invertebrate OR was identified through the determination of the whole genome of nematodes. In 1999, the first OR of an insect was identified in *Drosophila melanogaster* [9,10,11]. This OR is different from the G protein-coupled receptors of vertebrates; it is a macromolecular hydrophobic protein with seven transmembrane helical structures [12]. Insect ORs can be divided into two categories; one category is common ORs that bind odor molecules, and the other is olfactory receptor co-receptors (Orco) [13]. Orco interacts with ORs to form heterodimers (Orco/OR), resulting in functional ion channels that do not react directly to odorant substances but are directly activated by intracellular cAMP or cGMP; therefore, unlike the olfactory recognition pathway of mammalian G-protein-coupled receptors that rely on second messengers to activate ion channels, insect ORs can recognize odor more quickly and efficiently [14,15].

The insect genome contains OR genes with different functions, each of these ORs express a protein that binds to a specific ligand molecule at a specific site [16]. Coleoptera, the largest order of Insecta, has recently been found to express OR families of nearly forty species at the genome and transcriptome levels [17,18,19,20,21,22,23,24,25,26,27,28,29,30]. Most polyphagous insects show diversity in the number of OR genes among different species. Indeed, the correlation between the number of ORs and insect feeding habits suggests a potential connection between the diversity of ORs and the species’ ecological habitat and host range [19]. The rapid contraction and/or expansion of the OR family to adapt to complex and changing environments also increases the complexity of OR phylogenetic analysis and further limits our ability to predict the function of key ORs [19]. At present, only twelve ORs in Coleoptera, namely three in *Megaxylene Caryae* (*McarOR3, McarOR5,* and *McarOR20*), four in *Ips typographus* (*ItypOR5, ItypOR6, ItypOR46,* and *ItypOR49*), two in *Hylobius abietis* (*HabiOR3 and HabiOR4)*, two in *Dendroctonus ponderosae* (*DponOR8 and DponOR9*), and one in *Rhynchophorus ferrugineus* (*RferOR6*) have been assigned to effective ligand components and classified as functional ORs. *McarOR3*, *McarOR5,* and *McarOR20* are sensitive to components of aggregation pheromones released by males, including (S)-2-methyl-1-butanol, 2-phenylethanol and (25,3R)-2,3-hexanediol, while *ItypOR46* and *ItypOR49* recognize the male aggregation pheromone components of *I. typographus*, including ipsenol and ipsdienol [26,30]. *HabiOR3*, *DponOR8,* and *ItypOR6* respond exclusively to 2-phenylethanol, and *HabiOR4*, *DponOR9,* and *ItypOR5* respond to angiosperm green leaf volatiles [31]. *RferOR6* is narrowly tuned to alpha-pinene [32]. Combining the identification of gene family with the specific expression patterns of the gene to explore possible functional ORs can provide insights that facilitate more comprehensive screening of target genes for pest control and prevention.

*Anoplophora glabripennis* (Coleoptera: Cerambycidae) is a worldwide quarantine pest native to Asia. As a polyphagous insect, it has possible hosts in 15 families, 37 genera (e.g., *Acer*, *Populus*, *Salix,* and *Ulmus*). Larvae damage the xylem and phloem, which results in the decline of tree vigor and death, causing a decline in forest productivity and loss of forest resources [33]. *A. glabripennis* is primarily distributed in Asia. However, in 1996, *A glabripennis* was identified in Brooklyn, New York, NY, USA, and then rapidly colonized other parts of North America and Europe making it a quarantine pest worldwide [34,35]. After eclosion, the beetles feed on tender leaves of the host twigs for one week, and then gradually arrive at sexual maturation. Virgin male and female beetles gather together under the influence of both host plant volatiles and male aggregation pheromones [36]. Virgin males select suitable mates through vision and volatiles released by females and complete the mating process under the stimulation of female contact pheromones [37]. After mating, females sense the host plant volatiles to locate a suitable spawning site. Therefore, host plant volatiles and sex pheromones play an important role in regulating the breeding activities of beetles [38]. Previous electrophysiological and behavioral studies have reported that the volatiles released by the host plants of *A. glabripennis* include terpenes, alcohols, aldehydes, and acetates. The *A. glabripennis* male aggregation pheromones are mainly comprised of two hydroxyl ethers, and the female contact pheromones include five unsaturated long-chain hydrocarbons [39,40,41,42,43,44,45,46]. Attractant mixtures containing host plant volatiles and male aggregation pheromones have been used to trap *A. glabripennis* in forests, but with little success [47]. Previous studies have clarified the types of antennae receptors of beetles at different life stages. Olfactory sensory neurons located in the trichomes of the amphoteric antennal whipstock, for example, are more sensitive to the male-produced aggregation-sex pheromones 4-(n-heptyloxy)butan-1-ol and 4-(n-heptyloxy)butanal. Olfactory neurons of the conical sensilla of whipstock, on the other hand, have more obvious responses to alcohols, aldehydes, and terpenes, indicating that different types of *A. glabripennis* sensilla may have selective sensitivity to odor cues [48]. In a previous study, Hu et al. (2017) screened thirty-seven *AglaORs* on the *A. glabripennis* antennal transcriptome [49]. Mitchell et al. (2017) screened 121 *AglaORs* on the Agla1.0 genome, but they were not verified experimentally [25]. The four sex-biased *AglaORs* predicted in male and female whole-body transcriptomes are expressed at extremely low levels in antennae, suggesting that they might not be involved in the olfactory perception of volatile pheromone compounds (Zhang, unpublished data). Therefore, there has been no report of the key *AglaORs* involved in olfactory recognition in *A. glabripennis*. Later, the genome of *A. glabripennis* V2 version was published in U.S. DEPARTMENT OF AGRICULTURE (https://www.usda.gov/, accessed on 10 October 2022) by McKenna, which provided more accurate and complete genetic information. The Agla2.0 genome may help to identify members of the *AglaOR* gene family more comprehensively and provide reliable data for further screening of key genes related to smell.

In this study, ninety-eight candidate *AglaORs* were re-identified based on the Agla2.0 genome. We analyzed sequence characteristics, phylogenetic relationships, and gene and protein structures, to characterize in more detail the basic structural features of the gene family. In addition, the expression patterns of *AglaORs* in various sex tissues and at different developmental stages of the adult were analyzed by RT-qPCR. Moreover, key *AglaORs* participating in host localization or sexual communication were screened, providing a theoretical basis for elucidating the molecular mechanism of *A. glabripennis* olfaction and opening the perspective of identifying candidate target genes for pest control and prevention.

## 2. Results

### 2.1. Genome-Wide Identification of OR Genes in A. glabripennis

In order to obtain more comprehensive information on the *AglaORs* gene family, we first identified 127 candidate *AglaORs* based on the Agla2.0 genome using blastP and the Hidden Markov Model. After filtering out nineteen atypical odor receptors belonging to the 7tm-4 subfamily and ten repetitive short sequences encoding less than one hundred amino acids, ninety-eight genes were finally identified for analysis in this study (*AglaOR1-98*). Two genes (*AglaOR22/23*) were newly identified in this study and had sequence similarities with *AglaOR21* (XP_023311850.1) of 73.16% and 75.76%, respectively. Among the ninety-eight genes in the *AglaOR* gene family, fifty-eight genes had complete open reading frames encoding potentially functional proteins, and forty genes lacked 5’ or 3’ end structures. The full-length AglaORs had the potential to encode proteins of 302–540 amino acids, with molecular weights of 35.11–77.26 KDa, isoelectric points of 5.27–9.79, and grand average of hydropathicity values > 0. Subcellular localization showed that most of the AglaORs proteins were localized to the cell membrane (Table 1). These characteristics were consistent with the typical macromolecular hydrophobic transmembrane protein structures of insect ORs.

### 2.2. Phylogenetic Analysis of AglaORs

**Figure 1:**
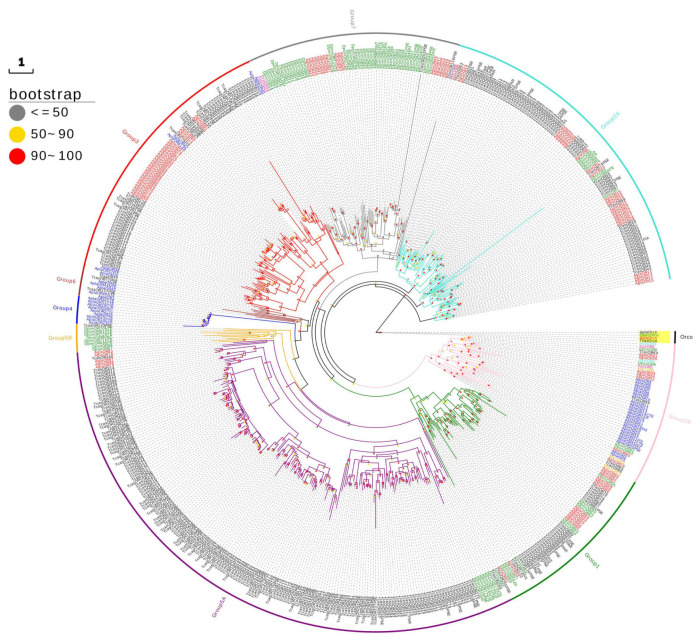
ORs of *Anoplophora glabripennis* (red), *Tribolium castaneum* (black), *Dendroctonus ponderosae* (green), *Agrilus planipennis* (blue), *Megaxylene Caryae* (orange), *Ips typographus* (pink), *Hylobius abietis* (Turquoise), and *Rhynchophorus ferrugineus* (purple) phylogenetic analysis. A phylogenetic tree was constructed according to the maximum likelihood method using PhyML (node support based on 1000 bootstrap replications is shown).

A phylogenetic tree was constructed using the OR gene families of *A. glabripennis*, *T. castaneum*, *D. ponderosae,* and *A. planipennis*, as well as ten functional ORs from *M. Caryae*, *I. typographus*, *H. abietis,* and *R. ferrugineus*. The results showed that the Orco of four species could be assembled into one single branch to form a clear homologous lineage. The OR of 573 other species formed different evolutionary branches and were divided into nine subfamilies, which is in line with the current phylogenetic map of the Coleoptera OR gene family (Figure 1). *T. castaneum* was mainly distributed in groups 5A, 2A, 1, and 3; *D. ponderosae* was mainly distributed in groups 7 and 5B; *A. planipennis* was mainly distributed in groups 4, 6, 2B; and ninety-seven ORs of *A. glabripennis* were mainly distributed in groups 3(27), 2A(26), 7(17), 1(14), 2B(7), and 5A(5) subfamilies. Genes within the same subfamily were also phylogenetically dispersed, suggesting that they evolved rapidly to accommodate wide ranges of hosts and to respond to different environmental conditions. In our phylogenetic results, *AglaOR29* was clustered with *MacrOR3*; *AglaOR32/34/35* were clustered with *MacrOR5*; *AglaOR61/62/64* were clustered with *MacrOR20*; *AglaOR9* was clustered with *RferOR6* and *AglaOR30/33* was clustered with the functional receptor group (*HabiOR3*, *DponOR8*, *ItypOR6*, *HabiOR4*, *DponOR9* and *ItypOR5*) in subfamily group 2B.

### 2.3. AglaOR Gene and Protein Structure

**Figure 2:**
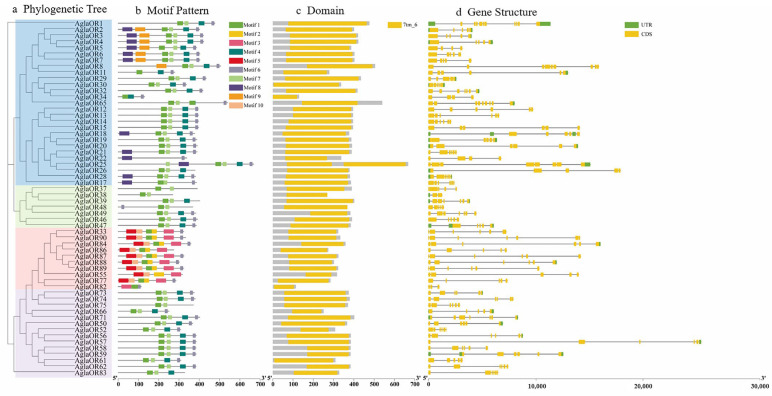
Phylogenetic relationship, conserved motifs, protein domains, and gene structure analysis of the AglaOR gene family. (**a**) Phylogenetic tree of AglaOR proteins. Different AglaOR subfamilies are indicated with different colors. Blue, Group I; green, Group II; red, Group III; and purple, Group IV. (**b**) Conserved motif analysis of AglaORs, with blocks of different colors representing different motif structures. (**c**) The protein domain of AglaORs, which belongs to the 7tm-6 subfamily. (**d**) Exon–intron structure of the AglaOR genes. Yellow boxes, black lines, and green boxes represent CDS, introns, and untranslated regions, respectively. (For interpretation of the color references in this figure legend, the reader is referred to the web version of this article.)

Comprehensive analysis of the conservative motif, conserved protein domain, and genetic structure of the AglaORs revealed differences in the motif structure between each subfamily (Figure 2). Details of the ten motifs are provided in the supplementary information. In Group I, motifs 1 and 6 were considered to be conserved motifs. Motifs 1 and 7 were unique motifs in Group II. Group III has motifs 1, 2, 3, 5, 6, and 10 as the conserved motif, AglaOR86 and AglaOR55 lacked motif 6, and AglaOR82 lacked motifs 2, 5, and 10. Group IV has motifs 1, 4, 6, and 7 as the conserved motif. The gene structure map of introns–exons showed that Group I mostly contains 3–7 introns, of which, AglaOR1 and AglaOR9 contain 10 and 9 introns, respectively. Most of Group II has 2–6 introns. Group III mostly contains 4–6 introns, except that of AglaOR82, which had two introns. Group IV mostly contains 4–6 introns.

### 2.4. Spatial–Temporal Differential Expression Analysis of AglaORs

#### 2.4.1. Analysis of Expression Pattern of AglaORs in Different Sex Tissues of Adults

**Figure 3:**
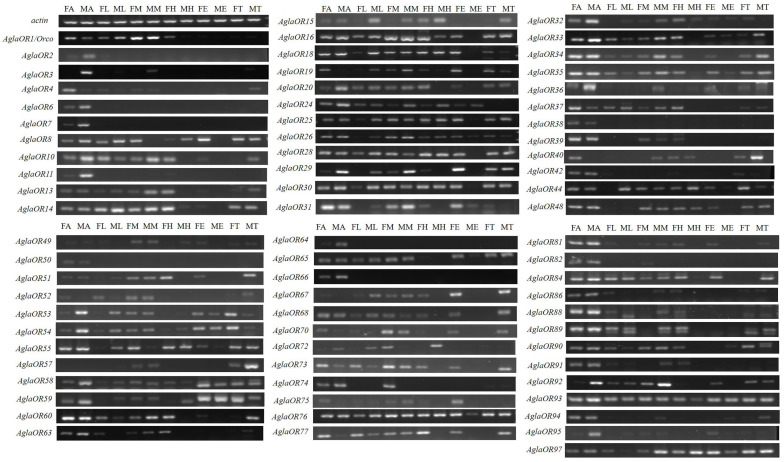
Expression profiles of *AglaORs* revealed by RT-PCR analysis of different adult tissues. FA, female antennae; MA, male antennae; FL, female leg; ML, male leg; FM, female mandibular whiskers; MM, male mandibular whiskers; FH, female head; MH, male head; FE, female external genitalia; ME, male external genitalia; FT, female thorax; MT, male thorax. *Actin* was used as the reference gene for each cDNA template. The intensity of the band is indicative of the expression level of the gene in different tissues.

By Sanger sequencing ninety-eight *AglaORs*, we filtered out twenty-six ORs that could not be amplified to the target sequence. The expression patterns of seventy-two *AglaORs* verified by sequencing in the antenna, leg, mandibular palps, head, external genitalia, and thorax of males and females were analyzed by RT-PCR. The results showed that although OR expression levels were low, they showed broad tissue expression profiles. Twenty-seven ORs were expressed in each tissue (*AglaOR8/10/13/14/16/18/20/25/26/28/30/37/44/48/53/54/55/58/59/65/67/68/76/77/84/89/90*). The expression levels of twenty-three ORs in amphoteric antenna were high (*AglaOR3/6/7/11/19/25/29/31/32/33/34/35/38/42/50/60/66/73/74/82/86/88/91*), and *AglaOR27/45/47/49* were highly expressed in the leg and mandibular palps (Figure 3).

**Figure 4:**
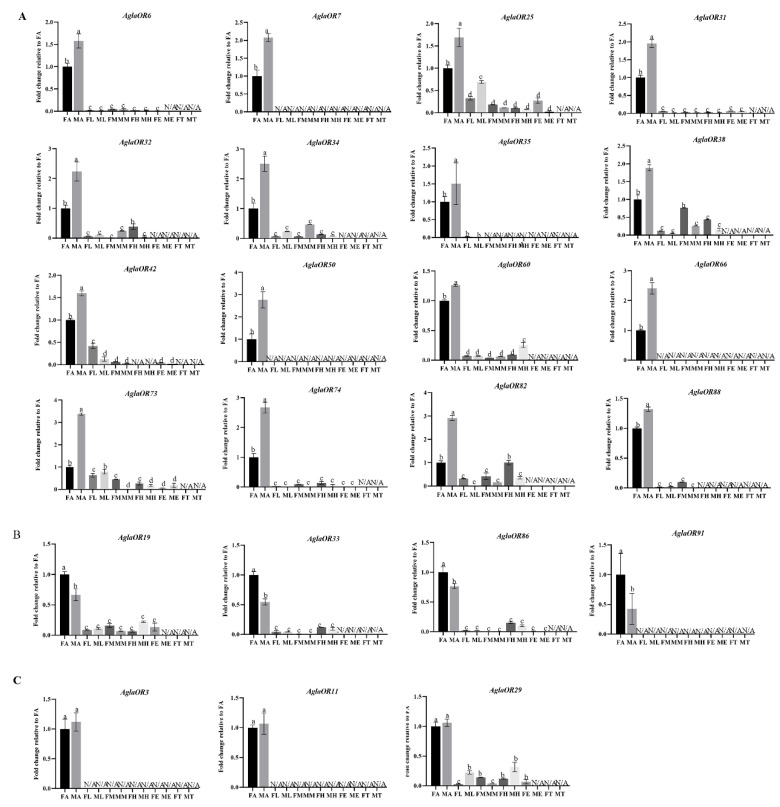
The expression levels of *AglaOR* genes in different sex tissues of adults analyzed by RT-qPCR. The gene expression of FA (female antennae) was used as the control. *Actin* was used as a housekeeping gene to normalize the expression level of each treatment. The relative expression levels represent mean ± standard error of the mean (SEM). Lowercase letters above the error bars indicate significant differences (*p* < 0.05, LSD). N/A means that the expression level is too low to be displayed. Subfigure (**A**) represents the genes with male antenna biased expression, (**B**) represents the genes with female antenna biased expression, and (**C**) represents the genes with no significant difference between two sexes.

To confirm the results of RT-PCR, we used RT-qPCR to conduct relative quantitative expression analysis of the twenty-three ORs with high antennal expression in different sex adult tissues. Sixteen ORs showed significant male-biased expression (*AglaOR6/7/25/31/32/34/35/38/42/50/60/66/73/74/82/88*) (Figure 4A); four ORs showed significant female-biased expression (*AglaOR19/33/86/91*) (Figure 4B); and three ORs showed significant antennal-biased expression (*AglaOR3/11/29*). However, there were no significant differences in expression between the sexes (Figure 4C).

#### 2.4.2. Analysis of Expression Patterns of AglaORs in Adults at Different Stages of Development

**Figure 5:**
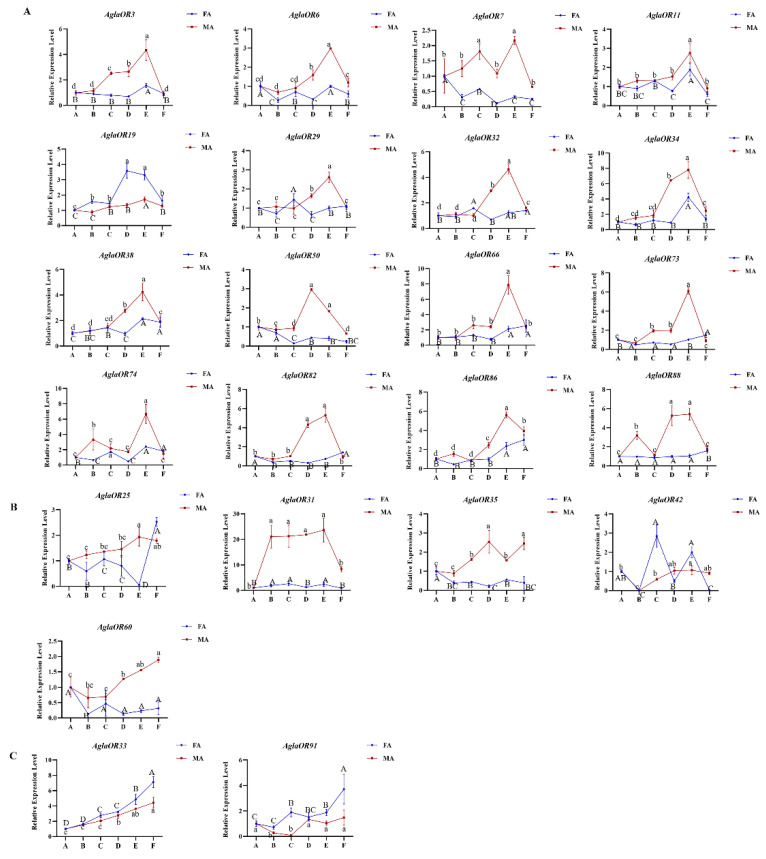
The expression levels of *AglaORs* at different stages of adult development were analyzed by RT-qPCR. A, eclosed in the pupal chamber; B, eclosed; C, received supplemental nutrition for 1 d; D, received supplemental nutrition for 7 d; E, received supplemental nutrition for 12 d; and F, post-mating. *Actin* was used as a housekeeping gene to normalize the expression level of each treatment. The gene expression of A (eclosion in pupal chamber) was used as the control. The relative expression level is expressed as the standard error (SEM) of the mean value. The lowercase letters above the bar indicate significant differences in FA expression at different developmental stages, and the uppercase letters indicate significant differences in MA expression at different developmental states (*p* < 0.05, LSD). Subfigure (**A**) represents the genes with significant high expression in sexual maturity, (**B**) represents the genes with no significant difference expression in each development stage, and (**C**) represents the genes with significant high expression after female post-mating grooving phase.

RT-qPCR analysis of the expression patterns of twenty-three ORs with high antennal expression in different developmental stages of *A. glabripennis* adults showed that sixteen ORs were significantly upregulated in the sexual maturation stage and significantly downregulated after mating (*AglaOR3/6/7/11/19/29/32/34/38/50/66/73/74/82/86/88*) (Figure 5A). *AglaOR3* also showed significantly higher expression after 1 d nutrient supplementation. *AglaOR33/91* was significantly overexpressed in the female post-mating grooving phase (Figure 5C). There were no differences in *AglaOR25/31/35/42/60* expression between different development stages (Figure 5B).

## 3. Discussion

Prediction of gene family members based on sequence similarity and protein conservative domains depends largely on the maturity of genomic assembly and the integrity of genomic information. Incomplete OR gene sequences may be related to problems in genomic assembly or sequencing methods, or to the rapid evolution of the OR gene family within complex environments consisting of a wide range of odor molecules. In this study, we found many incomplete OR gene sequences in the OR gene families of *T. castaneum*, *Rhaphuma horsfieldi*, *D. ponderosae*, *A. Planipennis*, *Aphis gossypii*, *Ambrostoma quadriimpressum*, and *Migratory locust* [18,23,28,50,51,52]. The ninety-eight *AglaORs* in this study, including thirty-seven *AglaOR* sequences identified by the antennal transcriptome [49], had ninety-six sequence matches compared to those identified by the Agla1.0 genome [25]. The members of OR gene family were obtained systematically and comprehensively, which will provide provides rich data for key gene mining.

Gene structure and conservative motif patterns are important for studying the evolutionary relationship of genes and the functional diversity of proteins in gene families. The conserved protein domain is important for OR structure, and the motif pattern can fine-tune the function of OR and lead to subtle differences in the binding of different odor molecules. We found that motifs 1, 6, and 7 are conserved motifs that are present in all genes and that they consist of 41, 16, and 11 amino acids, respectively. These motifs presumably play a conservative role in evolution. Motifs 2, 3, 5, and 10 are characteristic of subfamily Group III, which may regulate specific functions in this family. In the whole *AglaOR* gene family, the differences in the number and length of introns, which can affect gene expression, showed that they had been repeatedly acquired and lost [53,54]. AglaOrco (AglaOR1) had the most introns (ten), and the gene is highly expressed in olfactory tissues. The structure of the *AgosOrco* gene reported by Cao also has ten introns [50]. The single homologous *Orco* lineage can be fully explained by its conservation during evolution.

Using phylogenetic analysis, OR genes of the four species were assigned to nine subfamily lineages of the newly revised OR gene family of Coleoptera [19]. Notably, *AglaOR30/31/32/34/35* are homologous to seven functional genes that recognize 2-phenylethanol or angiosperm green leaf volatiles, and they belong to the 2B subfamily, in which *AglaOR31/32/34/35* exhibits male-antenna biased expression. It is speculated that these may be involved in the recognition of the above-mentioned compounds and we will conduct functional research on these genes in the future. There were no homologous genes in the *AglaOR* gene family that can be compared with the cluster of pheromone receptor Ityp46/49 of *I. typographus*. Ipsenol and ipsdienol are specific aggregation pheromone components of *IPs*. Males feeding on conifer species such as *Pinus* and *Picea* release a large amount of clastic pheromones, which signal male and female adults to hosts [55]. *A. glabripennis* selectively feeds on broad-leaved tree species. Therefore, differences in host selection may result in failure to evolve homologous pheromone receptors that recognize similar pheromone components.

Gene expression patterns are closely related to the function of encoded proteins. A typical OR is selectively expressed in olfactory neurons with low expression. In this study, RT-PCR analysis of the tissue expression profiles of *AglaORs* showed that the expression levels of twelve *AglaORs* were extremely low in all tissues. Low *AglaOR* expression may indicate that these genes exercise other functions, or that they are activated to recognize odorant molecules in other life states or under specific environmental conditions. The twenty-nine *AglaORs* were expressed in all tissues, indicating that they might also be involved in functions other than olfactory recognition, such as processing of olfactory bulb signals in the brain, detection of pheromone release, or regulating the reproductive process by affecting the development of sperm or egg cells [56,57]. Besides the antenna, the sensory organs such as the leg and mandibular palps also play a role in the perception of non-volatile compounds. For example, Hoover et al. (2014) reported that four sex-trace pheromones, 2-methyldocosane and (Z)-9-tricosene as major components and (Z)-9-pentacosene and (Z)-7-pentacosene as minor components, were action-labeled volatiles left by females [56]. It seems likely that the four highly expressed *AglaORs* in the leg or mandibular whisker (*AglaOR27/45/47/49*) are involved in the identification of contact and trace pheromones, and we propose that they could be used as important gene candidates to characterize further ligand binding for functional mining.

The antenna, which is the main organ used for detecting odor molecules in *A. glabripennis*, contains various types of receptors distributed on its surface. Odor molecules enter the sensillum lymph through the sensillum micropores and bind to ORs to activate the nerve center under the carriage of odorant-binding proteins (OBPs). Therefore, ORs specifically expressed in the antenna are considered to have an important function in recognizing odor molecules. Only 23 of the 98 *AglaORs* in the present study showed high antennal-biased expression, indicating the need to mine key genes using gene family identification in combination with gene expression pattern analysis. Among the 23 *AglaORs*, 20 showed significant male and female antennal-biased expression. Four *AglaORs* (*AglaOR19/33/86/91)* showed significant female antennal-biased expression. We speculate that they play a role in sensing male aggregation pheromones before mating or in finding spawning sites after mating, based on the analysis of expression patterns of adults at different stages of development. *AglaOR19/86* expression was significantly upregulated at sexual maturation and significantly downregulated after mating, indicating that this gene plays a role in sexual communication. Moreover, because pheromone release is inhibited during mating, the ability of these receptors to recognize the pheromone is weakened, leading to a decrease in their expression. *AglaOR33/91* expression was significantly upregulated in females searching for spawning sites after mating, suggesting that it might recognize host plant volatiles and determine the choice of spawning site. Another 16 *AglaORs* showed significant male antennal-biased expression, suggesting that these genes play an important role in the recognition of female pheromones prior to mating. We also found that the expression of eleven of the genes was significantly upregulated at sexual maturation and significantly downregulated after mating, suggesting that these genes are involved in premating sexual communication. Although the other five genes had male antennal-biased expression, they were not differentially expressed between the different development stages, suggesting that they might play an ongoing role in regulating olfaction. At the same time, we also found that *AglaOR3* was highly expressed at 1 d post-eclosion after feeding on the host. We speculate that this gene may be involved in the selection of the feeding host after eclosion. Although *AglaOR25* showed male antennae-biased expression, the expression in female antennae was significantly increased after mating. Thus, this gene may recognize a highly broad spectrum of odorants, allowing it to play different roles in various physiological states. After eclosion, *A. glabripennis* develops in the pupal chamber from 7–14 d, and then bites out of the circular eclosion hole and leaves the chamber [58]. However, here we found no significant difference in the expressed ORs between the two physiological states of eclosion in the pupal chamber and eclosion. We hypothesize that the beetle may rely on its tentacles and mouthparts to gnaw on the phloem and xylem of trees to explore the surrounding environment, and sensing odor molecules through the antennae may not dominate this process. Likewise, it is possible that candidate genes involved in this process have yet to be discovered, which is a direction for future research.

## 4. Materials and Methods

### 4.1. Identification and Sequence Analysis of the AglaOR Gene Family

#### 4.1.1. Identification of AglaOR Gene Family

Genomic information for *A. glabripennis* was obtained from the NCBI database (PRJNA167479). To identify *AglaOR* gene family members, the published OR proteins of *Drosophila melanogaster* and *T. castaneum* were used as queries to search against the genome of *A. glabripennis* using blastP with an E-value < 1 × 10^−5^. The HMM file for OR (PF02949:7tm odorant receptor) was then downloaded from the Pfam database. The 7tm-6 subfamily domain in the protein sequence of *A. glabripennis* was searched for using HMMER3.0 (E-value < 0.01) [59]. The presence of conservative domains in candidate proteins was confirmed using NCBI Preserved Domain Database (https://www.ncbi.nlm.nih.gov/Structure/cdd/wrpsb.cgi, accessed on 12 October 2022) and Smart Database (http://smart.embl-heidelberg.de/, accessed on 12 October 2022). The integration deleted the short sequence encoding less than 100 amino acids and confirmed the complete *AglaOR* gene family. All *AglaORs* sequences were analyzed by EXPASY (https://wolfpsort.hgc.jp/, accessed on 12 October 2022) to obtain amino acid number (AA), molecular weight (MW), isoelectric point (PI), and transmembrane domain (TM). The sequences were also subjected to protein avidity/hydrophobicity analysis (GRAVY). Subcellular localization was predicted by WOLF PSORT (https://wolfpsort, accessed on 12 October 2022).

#### 4.1.2. Construction of Phylogenetic Tree

The OR gene family sequences identified from *T. castaneum*, *Dendroctonus ponderosae*, and *Agrilus planipennis* genomes were selected for participation in the construction of a phylogenetic tree to determine the branch positions of *AglaORs*, and ten functional Ors from *M. Caryae*, *I. typographus*, *H. abietis*, and *R. ferrugineus* were also included to determine whether they are homologous with *AglaORs* [18,22,23,31,32]. Maximum likelihood phylogenies were inferred using IQ-TREE [60] under a model automatically selected by IQ-TREE (‘Auto’ option in IQ-TREE) for 1000 ultrafast [61,62] bootstraps, as well as the Shimodaira–Hasegawa-like approximate likelihood-ratio test. Evolview (http://www.evolgenius.info/evolview, accessed on 13 October 2022) was used to visualize the evolutionary tree.

#### 4.1.3. Structural Characteristics Analysis of AglaORs

The gene structure information was extracted from the annotation file of *A. glabripennis* genome and TBtools (v1.09851) was used for visual analysis of the exon–intron structure of *AglaORs* [63]. The parameters were as follows: number of repetitions = arbitrary, maximum base number = 10, and optimal base width = 10–50 residues. The online tool MEME (http://meme-suite.org/, accessed on 17 October 2022) was used to perform a motif analysis of *AglaORs*. The structure of 10 motifs are shown in Figure S1. The conservative domain of *AglaORs* was analyzed by NCBI Preserved Domain Database (https://www.ncbi.nlm.nih.gov/Structure/bwrpsb/bwrpsb.cgi, accessed on 17 October 2022) with search mode = automatic, E-value < 0.01, and maximum number of hits = 500.

### 4.2. Analysis of Expression Characteristics of AglaORs

#### 4.2.1. Insect Collection and Processing

The natural poplar tree segments damaged by *A. glabripennis* were collected from Sanhe Forest Farm, Qingshui Town, Suzhou District, Jiuquan City, Gansu Province, China (39°34′ N, 99°10′ E). The sections were sealed with wax and shipped back to Beijing Laboratory for placement in a self-made wire mesh cage with dimensions 3 m × 3 m × 3 m. The indoor temperature was controlled at 25 ± 1 °C, and the relative humidity was within 60–70%. Tissues from both sexes of *A. glabripennis* were dissected using sterilized scissors and forceps, including the antenna, leg, maxillary palps, head (without maxillary palps), external genitalia, and thorax. Samples of male and female adults that were eclosed in the pupal chamber, eclosed and received supplemental nutrition for 1 d, 7 d, and 12 d, were collected. The reproductive organs gradually developed to sexual maturity 7–12 d after the beetles were supplemented with food. The males and females supplemented with food for 12 d were placed in insect feeding boxes in pairs and were collected after mating during female grooving behavior. Antennae of males and females are collected in different development stages mentioned above. The above process was repeated three times for each sample. Samples were frozen at −80 °C until use.

#### 4.2.2. RNA Extraction and RT-qPCR Analysis

Total RNA from each tissue was extracted using TRIzol reagent (No. 15596026; Invitrogen, Carlsbad, CA, USA) and RNeasy Plus Mini Kit (No. 74134; Qiagen, Hilden, Germany) according to the manufacturer’s instructions. RNA purity, concentration, and integrity were assessed by NanoDrop 8000 (Thermo, Waltham, MA, USA) and agarose gel electrophoresis. PrimeScript RT Reagent Kit with gDNA Eraser (No. RR047A; TaKaRa, Dalian, China) was used to extract 1 μg of total RNA for cDNA synthesis. Primer3Plus (http://www.primer3plus.com/cgi-bin/dev/primer3plus.cgi, accessed on 18 October 2022) was used to develop specific primers for RT-PCR and RT-qPCR. Primers were designed and sent to Beijing Ruiboxingke Biotechnology Co., Ltd. for synthesis.RT-PCR specific primer sequences are shown in Table S1, RT-qPCR specific primer sequences are shown in Table S2. RT-PCR was performed using a 2×Taq PCR Master Mix (No. BN12045; Biorigin, Beijing, China). A 12.5 μL system was used for each PCR reaction, including 2×Taq PCR Master Mix, 1 μL per primer pair, 1 μL cDNA template, and 9.5 μLd2H2O. The amplification procedure was as follows: 94 °C for 1 min and 30 s; followed by 34 cycles of 94 °C for 20 s; 52 °C for 20 s; 72 °C for 30 s; 72 °C for 5 min; and 4 °C indefinitely. The PCR products were subjected to 1.2% agarose gel electrophoresis to examine gene expression (voltage 120 V, 30 min, 1× TAE as electrophoresis buffer). PCR products with bright and single bands were selected for Sanger sequencing to remove the false-positive gene. RT-qPCR was performed using the Bio-Rad CFX96 PCR system (Hercules, CA, USA) and SYBR Premix Ex Taq II (No. RR820A; TaKaRa, Dalian, China). Each PCR reaction was conducted in 12.5 μL of reaction mixture containing 6.25 μL of SYBR Premix Ex Taq II, 0.5 μL of each primer, 1 μL of cDNA template, and 4.25 μL of ddH2O. The amplification procedure was as follows: 95 °C for 30 s, followed by 40 cycles of 95 °C 0.05 s, 60 °C 30 s, and 95 °C for 10 s, followed by increments of 0.5 °C from 65–95 °C for 0.05 s each to generate a dissolution curve. Actin commonly used for tissue expression of *A. glabripennis* was selected as the internal reference gene to normalize the expression level [64,65,66]. Three biological replicates and three technical replicates were performed. The relative expression amount was calculated based on the 2^−ΔΔ^Ct method [67].

#### 4.2.3. Data Analysis

One-way analysis of variance and least significant difference (LSD) tests were performed using SPSS 19.0 (IBM SPSS, Armonk, NY, USA). *p* < 0.05 indicated that the difference was statistically significant. The gene expression levels of female antennae were used as controls in the tissue expression profile, and the gene expression level at eclosed in the pupal chamber was used as the control for producing spatial–temporal expression profiles. Quantitative data were expressed as the standard error of the mean (SEM).

## 5. Conclusions

In this study, we identified the *AglaOR* gene family, constructed a phylogenetic tree, analyzed the structural characteristics of the gene proteins, and found that the *AglaOR* family exhibited structural and functional diversity. Using spatial–temporal differential expression analysis, twenty-three highly expressed *AglaOR* genes in the antenna were screened, revealing the key candidate genes involved in the premating sexual communication process (eleven male antennal-biased and two female antennal-biased) and the selection of female spawning grounds after mating (two female antennal-biased). To determine whether these genes have an olfactory recognition function and to assess their binding characteristics and sensitivity to ligand molecules, it is necessary to further explore genes using the xenopus oocyte/voltage clamp method. A comprehensive and systematic analysis of the OR gene family of *A. glabripennis* could provide a theoretical basis for further elucidating the molecular mechanism of olfactory recognition. The use of RNAi or CRISPR/Cas9 and other techniques to silence or knock out key *AglaORs* could be used to interfere with *A. glabripennis* olfaction, providing strategies for pest control and prevention over small environmental ranges.

## Figures and Tables

**Table 1:** Summary information of OR gene family in *Anoplophora glabripennis.*

GeneName	GeneID	CDS (bp)	Amino Acid Residues	Status	Molecular Weight (KDa)	Isoelectric Points	Grand Average of Hydropathicity	Transmembrane Helices	Subcellular Localization
AglaOR1	XP_018568191.1	1431	477	complete ORF	53.81	7.73	0.19	7	Endoplasmic reticulum
AglaOR2	XP_018560835.2	1203	401	complete ORF	46.93	8.49	0.318	7	Plasma membrane
AglaOR3	XP_023313104.1	1263	421	complete ORF	49.13	8.64	0.483	7	Plasma membrane
AglaOR4	XP_018560865.2	1266	422	complete ORF	49.49	8.88	0.495	7	Plasma membrane
AglaOR5	XP_023313097.1	1161	387	complete ORF	45.03	9.14	0.31	6	Plasma membrane
AglaOR6	XP_023313106.1	1209	403	complete ORF	47.3	8.88	0.331	7	Plasma membrane
AglaOR7	XP_023313105.1	1209	403	complete ORF	47.44	8.79	0.276	7	Plasma membrane
AglaOR8	XP_023310030.1	1515	505	complete ORF	58.08	6.6	0.08	7	Plasma membrane
AglaOR9	XP_018575345.1	555	185	5’ lost	21.81	7.8	0.349	3	Cytoskeleton
AglaOR10	XP_023310521.1	855	285	5’ lost	33.66	9	0.189	5	Cytoskeleton
AglaOR11	XP_018567483.1	1152	384	complete ORF	44.49	6.6	0.235	4	Cytoskeleton
AglaOR12	XP_023310034.1	1188	396	complete ORF	45.55	8.03	0.361	4	Plasma membrane
AglaOR13	XP_023310033.1	1188	396	complete ORF	45.89	6.15	0.416	5	Plasma membrane
AglaOR14	XP_018566518.1	1188	396	complete ORF	45.49	5.75	0.51	5	Plasma membrane
AglaOR15	XP_018566530.1	1188	396	complete ORF	45.54	5.99	0.42	5	Plasma membrane
AglaOR16	XP_023309742.1	693	231	5’ lost	26.47	7.81	0.49	4	Plasma membrane
AglaOR17	XP_018566967.1	1158	386	complete ORF	44.08	8.85	0.398	7	Plasma membrane
AglaOR18	XP_018569520.1	1131	377	complete ORF	43.78	8.49	0.307	5	Mitochondrion
AglaOR19	XP_018568462.1	1164	388	complete ORF	44.95	7.94	0.351	5	Plasma membrane
AglaOR20	XP_023313058.1	1173	391	complete ORF	45.37	6.05	0.431	5	Plasma membrane
AglaOR21	XP_023311850.1	1167	389	complete ORF	45.27	7.54	0.427	6	Plasma membrane
AglaOR22	XP_023311848.1	1014	338	complete ORF	38.8	8.13	0.205	4	Plasma membrane
AglaOR23	XP_023312636.1	699	233	3’ lost	26.98	6.01	0.309	2	Plasma membrane
AglaOR24	XP_023310658.1	759	253	3’ lost	29.91	8.65	0.211	5	Cytoskeleton
AglaOR25	XP_023311847.1	1041	347	complete ORF	77.26	7.44	0.372	10	Plasma membrane
AglaOR26	XP_023311849.1	1137	379	complete ORF	43.84	6.31	0.377	6	Plasma membrane
AglaOR27	XP_023309851.1	600	200	5’ lost	23.12	4.85	0.315	3	Plasma membrane
AglaOR28	XP_018564120.1	1143	381	complete ORF	44.27	8.11	0.397	7	Plasma membrane
AglaOR29	XP_018564808.2	1305	435	complete ORF	51.09	8.36	0.32	7	Cytoskeleton
AglaOR30	XP_018575063.1	1011	337	complete ORF	38.7	9.03	0.258	5	Cytoskeleton
AglaOR31	XP_018560873.1	861	287	5’ lost	32.1	8.64	0.365	6	Plasma membrane
AglaOR32	XP_018577142.1	1254	418	complete ORF	48.37	8.14	0.409	6	Plasma membrane
AglaOR33	XP_023311544.1	969	323	complete ORF	37.69	7.33	0.505	5	Plasma membrane
AglaOR34	XP_023310447.1	1263	421	complete ORF	14.9	8.47	0.617	1	Plasma membrane
AglaOR35	XP_023310446.1	390	130	5’ lost	14.73	7.12	0.58	3	Extracell
AglaOR36	XP_023310818.1	315	105	5’ lost	12.28	5.75	0.523	1	Plasma membrane
AglaOR37	XP_018570370.1	1170	390	complete ORF	45.77	9.55	0.167	6	Mitochondrion
AglaOR38	XP_023312982.1	1185	395	complete ORF	31.64	5.63	0.35	4	Extracell
AglaOR39	XP_023309827.1	1206	402	complete ORF	47.2	5.27	0.397	6	Plasma membrane
AglaOR40	XP_023312071.1	894	298	3’ lost	35.08	6.24	0.286	3	Plasma membrane
AglaOR41	XP_023310498.1	774	258	3’ lost	29.93	9.27	0.316	4	Plasma membrane
AglaOR42	XP_018567067.2	612	204	5’ lost	24.02	8.95	0.362	2	Plasma membrane
AglaOR43	XP_023310496.1	372	124	5’ lost	14.42	5.76	0.591	1	Plasma membrane
AglaOR44	XP_018577261.2	534	178	5’ lost	20.5	7.19	0.213	2	Plasma membrane
AglaOR45	XP_018562952.1	603	201	5’ lost	22.77	5.58	0.42	2	Plasma membrane
AglaOR46	XP_023311401.1	1173	391	complete ORF	45.43	6.24	0.437	6	Endoplasmic reticulum
AglaOR47	XP_018569507.1	1155	385	complete ORF	44.69	6.78	0.329	6	Plasma membrane
AglaOR48	XP_023313160.1	1104	368	complete ORF	43.55	9.16	0.285	6	Cytoskeleton
AglaOR49	XP_018571376.1	1149	383	complete ORF	45.21	9.18	0.311	6	Mitochondrion
AglaOR50	XP_018570955.1	1101	367	complete ORF	42.11	7.72	0.469	6	Plasma membrane
AglaOR51	XP_023309856.1	345	115	5’ lost	13.11	8.19	0.731	2	Cytoskeleton
AglaOR52	XP_018578983.2	924	308	complete ORF	35.1	8.58	0.212	4	Cytoskeleton
AglaOR53	XP_023310132.1	450	150	5’ lost	17.36	9.5	0.255	3	Plasma membrane
AglaOR54	XP_018578651.1	804	268	5’ lost	30.51	9.2	0.484	4	Plasma membrane
AglaOR55	XP_023311538.1	951	317	complete ORF	36.83	7.31	0.52	6	Plasma membrane
AglaOR56	XP_018579026.2	1155	385	complete ORF	43.78	7.8	0.502	7	Plasma membrane
AglaOR57	XP_018579015.2	1155	385	complete ORF	43.29	7.07	0.543	7	Plasma membrane
AglaOR58	XP_018567969.1	1152	384	complete ORF	44.31	9.79	0.25	7	Plasma membrane
AglaOR59	XP_023313053.1	1152	384	complete ORF	44.05	9.49	0.268	7	Plasma membrane
AglaOR60	XP_023309848.1	735	245	5’ lost	29.07	8.64	0.134	2	Cytoskeleton
AglaOR61	XP_018578867.1	927	309	complete ORF	35.54	8.43	0.267	5	Cytoskeleton
AglaOR62	XP_023310463.1	1152	384	complete ORF	44.41	6.43	0.321	6	Plasma membrane
AglaOR63	XP_018560823.2	624	208	5’ lost	24.3	9.13	0.399	3	Plasma membrane
AglaOR64	XP_023310462.1	612	204	5’ lost	23.56	6.5	0.426	3	Plasma membrane
AglaOR65	XP_023311417.1	1620	540	complete ORF	62.83	8.64	0.362	6	Plasma membrane
AglaOR66	XP_023312511.1	1215	405	complete ORF	46.23	8.4	0.355	2	Plasma membrane
AglaOR67	XP_023310753.1	822	274	5’ lost	31.76	7.69	0.333	4	Plasma membrane
AglaOR68	XP_023310752.1	822	274	5’ lost	31.58	6.24	0.452	4	Plasma membrane
AglaOR69	XP_023309980.1	507	169	5’ lost	19.42	8.58	0.304	1	Cytoskeleton
AglaOR70	XP_018561943.1	660	220	5’ lost	25.16	7.8	0.161	1	Mitochondrion
AglaOR71	XP_023312510.1	1206	402	complete ORF	46.2	6.77	0.334	6	Plasma membrane
AglaOR72	XP_018575789.2	810	270	5’ lost	30.38	6.12	0.303	3	Plasma membrane
AglaOR73	XP_018561953.2	1125	375	complete ORF	43.83	7.52	0.377	5	Plasma membrane
AglaOR74	XP_023310232.1	1140	380	complete ORF	44.34	6.72	0.285	4	Plasma membrane
AglaOR75	XP_023309981.1	1113	371	complete ORF	42.52	8.52	0.505	4	Plasma membrane
AglaOR76	XP_018568489.1	318	106	5’ lost	12.68	6.22	0.154	0	Plasma membrane
AglaOR77	XP_023309849.1	1155	385	complete ORF	33.44	8.73	0.541	4	Plasma membrane
AglaOR78	XP_018563443.1	588	196	5’ lost	22.71	8.93	0.196	2	Plasma membrane
AglaOR79	XP_023309850.1	552	184	5’ lost	21.39	9.05	0.207	0	Cytoskeleton
AglaOR80	XP_018560827.2	564	188	5’ lost	21.94	9.56	0.282	2	Nucleus
AglaOR81	XP_023309854.1	501	167	5’ lost	19.2	8.45	0.334	3	Plasma membrane
AglaOR82	XP_023312904.1	1080	360	complete ORF	41.71	8.75	0.467	2	Plasma membrane
AglaOR83	XP_018570369.1	984	328	complete ORF	38.23	7.73	0.514	4	Plasma membrane
AglaOR84	XP_023309845.1	1077	359	complete ORF	41.76	8.51	0.463	4	Plasma membrane
AglaOR85	XP_023309844.1	882	294	5’ lost	34.42	7.39	0.488	4	Plasma membrane
AglaOR86	XP_023309852.1	1029	343	complete ORF	39.98	7.5	0.52	4	Plasma membrane
AglaOR87	XP_023311541.1	972	324	complete ORF	37.55	6.35	0.539	5	Plasma membrane
AglaOR88	XP_023309846.1	906	302	complete ORF	35.1	8.32	0.497	4	Plasma membrane
AglaOR89	XP_023309847.1	969	323	complete ORF	37.45	7.68	0.557	5	Plasma membrane
AglaOR90	XP_023311539.1	999	333	complete ORF	38.51	7.26	0.471	4	Plasma membrane
AglaOR91	XP_023311540.1	771	257	5’ lost	29.63	8.69	0.038	2	Cytoskeleton
AglaOR92	XM_023456081.1	1206	402	5’ lost	42.21	6.38	0.394	5	Plasma membrane
AglaOR93	XM_018713238.2	529	176	5’ lost	10.87	8.78	0.484	0	Extracell
AglaOR94	XM_023454061.1	775	258	5’ lost	12.21	5.24	0.679	1	Extracell
AglaOR95	XM_018723466.1	768	256	5’ lost	9.04	43.98	0.226	7	Plasma membrane
AglaOR96	XM_018712442.2	1248	416	5’ lost	7.96	44.72	0.429	7	Plasma membrane
AglaOR97	XM_018712974.1	832	277	5’ lost	8.63	44.81	0.378	6	Endoplasmic reticulum
AglaOR98	XM_018712973.1	832	277	5’ lost	9.28	44.92	0.367	6	Plasma membrane

## Data Availability

All data mentioned in this paper are available at the National Center for Biotechnology Information (NCBI) with the BioProject no. PRJNA167479. The reference sequences of the phylogenetic tree are detailed in the references in the methods section.

## Supplementary Materials

The supporting information can be downloaded at: https://www.mdpi.com/article/10.3390/ijms24021625/s1.
