# Supplementary figures and images for: Genome-Wide Identification of the Odorant Receptor Gene Family and Revealing Key Genes Involved in Sexual Communication in Anoplophora glabripennis
Source: Int J Mol Sci. 2023 Jan 13;24(2):1625. doi: 10.3390/ijms24021625 (PMC9861320; doi:10.3390/ijms24021625)

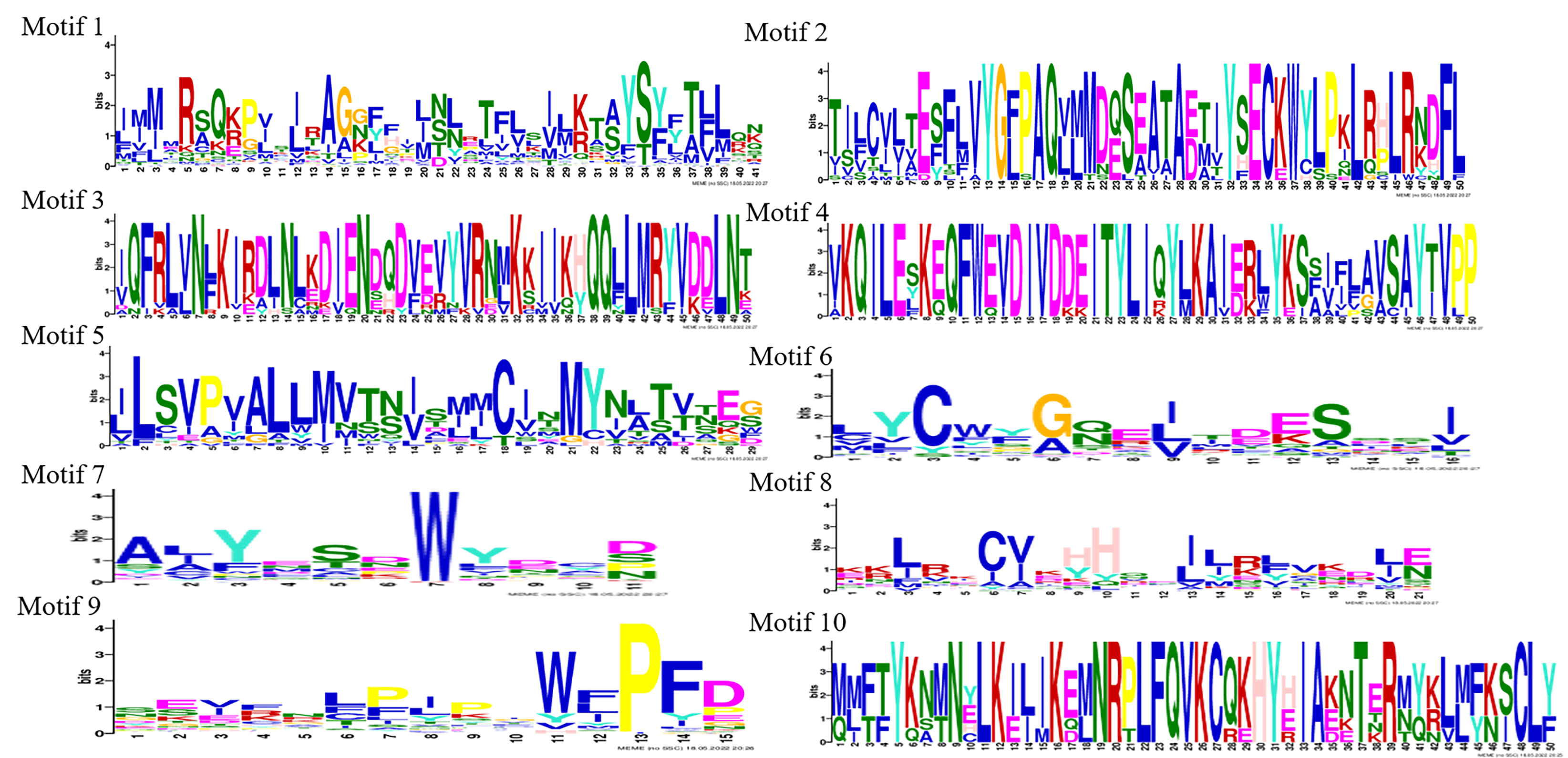

Supplement: Supplementary file 1 [file ijms-24-01625-s001.zip › FigureS1.tif]
